# Risankizumab in Japanese patients with moderate‐to‐severe palmoplantar pustulosis: Results from the randomized, phase 3 JumPPP study

**DOI:** 10.1111/1346-8138.17659

**Published:** 2025-02-25

**Authors:** Yukari Okubo, Masamoto Murakami, Satomi Kobayashi, Shigeyoshi Tsuji, Mitsumasa Kishimoto, Kimitoshi Ikeda, Maiko Jibiki, Ezequiel Neimark, Byron Padilla, Jie Shen, Sydney Peters, Tadashi Terui

**Affiliations:** ^1^ Department of Dermatology Tokyo Medical University Hospital Tokyo Japan; ^2^ Department of Dermatology Ehime University School of Medicine Tōon Japan; ^3^ Department of Dermatology Seibo International Catholic Hospital Tokyo Japan; ^4^ Department of Rehabilitation, Orthopedics and Psoriasis Center Nippon Life Hospital Osaka Japan; ^5^ Department of Nephrology and Rheumatology Kyorin University School of Medicine Tokyo Japan; ^6^ AbbVie GK Tokyo Japan; ^7^ AbbVie Inc North Chicago Illinois USA; ^8^ Division of Dermatological Science, Department of Dermatology Nihon University School of Medicine Tokyo Japan

**Keywords:** Japan, Japanese, palmoplantar pustulosis, psoriasis, risankizumab

## Abstract

Palmoplantar pustulosis (PPP) is a chronic, debilitating skin disease of the palms and/or soles. We report the efficacy and safety of risankizumab (RZB), an interleukin 23 p19 inhibitor, from the JumPPP study (a phase 3, multicenter, randomized, placebo‐controlled, double‐blind study to evaluate RZB in adult Japanese sUbjects with Moderate‐to‐severe PalmoPlantar Pustulosis; NCT04451720). Patients were randomized 1:1 to receive RZB (150 mg) or placebo at weeks 0 and 4; all patients received RZB from week 16 to week 52 (patients initially randomized to RZB) or week 56 (patients initially randomized to placebo). The primary end point was a Palmoplantar Pustulosis Area and Severity Index (PPPASI) change from baseline; secondary end points were ≥50%/≥75% improvement in PPPASI (PPPASI 50/75) at week 16. Efficacy and safety were evaluated to 68 and 76 weeks, respectively. In total, 119 patients (RZB, *n* = 61; placebo, *n* = 58) were enrolled. Greater improvement with RZB versus placebo was demonstrated by the significant difference in PPPASI change from baseline at week 16 (least squares mean treatment difference, −3.48; *p* < 0.05). At week 16, a greater proportion of patients receiving RZB vs placebo achieved PPPASI 50 (41.0% vs 24.1%; nominal *p* < 0.05) but not PPPASI 75 (13.1% vs 15.5%; nominal *p* = 0.74). Improvements generally continued through to week 68. The safety profile was generally consistent with previous studies of RZB in psoriasis. RZB demonstrated efficacy over placebo at week 16 in Japanese patients with PPP, with improvements sustained through to week 68, and was well tolerated with no unexpected safety findings.

## 1 INTRODUCTION

Palmoplantar pustulosis (PPP) is a chronic, debilitating, skin disease of the palms and/or soles characterized by repeated episodes of exacerbation and remission.^1^, ^2^ PPP predominantly presents in women, and patients often have a history of smoking and focal infection.^3^, ^4^ The prevalence of PPP in the Japanese population is estimated to be 0.12%, which is higher than the prevalence reported in other developed countries (USA, 0.009%; Denmark, 0.005%; Germany, 0.08%).^4^, ^5^ Pustulotic arthro‐osteitis (PAO) is a major comorbidity that affects up to 30% of patients with PPP (predominantly women). PAO commonly involves the sternocostoclavicular region and is typically associated with pain in the anterior chest; it may be accompanied by pain in other joints.^6^ With or without PAO, PPP may place a substantial burden on patients' quality of life, ultimately leading to considerable impact on functional disability.^7^, ^8^ Thus, assessments of quality of life and physical function outcomes are of great importance and should include evaluations of pain, discomfort, and disability.

Palmoplantar pustulosis is a challenging disease to manage, and current treatment modalities include topical drugs, systemic drugs, and phototherapy.^9^ Corticosteroids and vitamin D3 drugs are common topical treatments but show limited efficacy, perhaps due to the thick stratum corneum acting as a barrier to palm and sole lesions. Additionally, long‐term use of topical corticosteroids can lead to undesirable adverse reactions on the applied regions, such as skin atrophy, capillary dilation, and infection. Chronic inflammation may be linked to the pathogenesis of the interleukin (IL)‐23 axis. IL‐23 expression is increased in PPP lesions, making research into the impact of IL‐23 inhibition of special interest for treating PPP.^10^ Agents that target the IL‐23^11^, ^12^ and IL‐17 pathways^13^ have shown efficacy in randomized, placebo‐controlled studies in PPP.

Risankizumab (RZB) is a humanized immunoglobin G1 monoclonal antibody that specifically inhibits IL‐23 by binding to its p19 subunit. It is approved in Japan to treat psoriasis vulgaris, psoriatic arthritis, generalized pustular psoriasis, erythrodermic psoriasis, and PPP in adults.^14^ The efficacy of RZB in patients with psoriasis has been demonstrated in clinical trials;^15^, ^16^, ^17^, ^18^, ^19^, ^20^ however, the efficacy of RZB for the treatment of PPP in randomized, placebo‐controlled clinical trials has not been published. Here, we report the primary and long‐term efficacy and safety data of RZB in treating Japanese adults with PPP.

## 2 METHODS

### 2.1 Patients

Eligible patients were aged 18 years or older who had moderate or severe PPP, with or without PAO, and who met the following criteria: Palmoplantar Pustulosis Area and Severity Index (PPPASI) total score of ≥12 (possible PPPASI total scores range from 0 [no sign of disease]–72 [very severe disease]); moderate or severe pustules/vesicles on at least one palm or sole (PPPASI severity score ≥2); inadequate/intolerant response to topical corticosteroids, vitamin D3 derivative preparations, phototherapy, and/or systemic etretinate; and stable disease activity (≤5‐point improvement in PPPASI total score during screening). Eligible patients must have completed focal infection procedures (e.g., tonsillectomy, dental therapy) 24 weeks before the first study drug dose; patients could be enrolled if they did not require a focal infection procedure as long as follow‐up care was appropriate. Confirmatory magnetic resonance imaging was performed before baseline in patients with suspected PAO that had not been previously confirmed. Patients were excluded from the study if they had plaque‐type psoriasis, pustular psoriasis, or drug‐induced PPP; an active or suspected malignancy or history of malignancy within 5 years before screening; acute infections including viral hepatitis, HIV, and/or tuberculosis; acute onset of exacerbation of chronic or recurrent focal infection within 1 month before the baseline visit; an active or suspected malignancy or history of malignancy within 5 years before screening; any other relevant medical conditions (e.g., cerebrovascular accident or myocardial infarction within 6 months before screening, organ transplant, chronic alcohol or drug abuse, history of hypersensitivity to study drug and/or other biologics in the same class); or active skin disease other than PPP that could potentially interfere with the trial assessments.

### 2.2 Study design and treatment

JumPPP (NCT04451720) was a phase 3, multicenter, randomized, placebo‐controlled, parallel‐group, double‐blind study evaluating the safety and efficacy of RZB in adult Japanese sUbjects with Moderate‐to‐severe PalmoPlantar Pustulosis. The study was conducted at 39 sites located in Japan. Patients were randomized using interactive response technology; randomization was stratified by baseline PPPASI total score (≤20, >20–30, >30) and baseline smoking status (yes, no). The investigator, study site personnel, and patient were blinded to the assigned treatment; prefilled RZB and placebo syringes were identical in appearance. In the double‐blind period (Period A; week 0–week 16), patients were randomized 1:1 to receive subcutaneously administered RZB (150 mg) or placebo injections at weeks 0 and 4. In the RZB‐treatment period (Period B; week 16 to week 56), both groups received RZB and placebo at different intervals while maintaining the study's blinding. Patients initially randomized to receive RZB continued to receive RZB at weeks 16, 28, 40, and 52 (with matching placebo at weeks 20, 32, 44, and 56), and patients initially randomized to receive placebo received RZB at weeks 16, 20, 32, 44, and 56 (with matching placebo at weeks 28, 40, and 52). The last study visit was at week 68; a follow‐up safety telephone call occurred 8 weeks after the last study visit. Written informed consent was obtained from all patients before enrollment.

### 2.3 Assessments

#### 2.3.1 Efficacy

The primary end point was the change in PPPASI total score from baseline to week 16. Secondary end points included the proportion of patients achieving ≥50% and ≥75% improvement in PPPASI (PPPASI 50/75) at week 16. Other efficacy end points assessed through to week 68 included PPPASI 50/75 and change from baseline in PPPASI total score, worst pruritus numeric rating scale (WP‐NRS) score, and Dermatology Life Quality Index (DLQI) score. Among patients with baseline PAO, changes from baseline in the modified Bath Ankylosing Spondylitis Disease Activity Index (mBASDAI) total score and mBASDAI anterior chest pain subscore to week 68 were also assessed.

Physical activity analyses (percentage change from baseline in daily step count and moderate‐to‐vigorous physical activity [MVPA] during weeks 1–16 and weeks 41–52) were assessed in the overall population, among patients who were sedentary (taking ≤5000 daily steps) at baseline, and among patients with or without PAO. Daily step count and minutes of MVPA were collected using a wrist‐based wearable device (CentrePoint Insight Watch, ActiGraph). The accelerometer sensor within the watch recorded continuous acceleration at 32 Hz. The resultant continuous data were processed using the UWFv1 algorithm to yield daily step counts and the Freedson Adult algorithm to yield daily minutes of MVPA.^21^, ^22^ The daily physical activity data were then averaged weekly after filtering out data from non‐compliant days (defined as <8 hours of wear). Data for weeks with less than 3 days of compliant wear were not included and considered as missing.

#### 2.3.2 Safety

Safety evaluations included monitoring of treatment‐emergent adverse events (TEAEs), serious adverse events (AEs), physical examinations, changes in vital signs and clinical laboratory tests, and local tolerability. Safety findings were tabulated using the Medical Dictionary for Regulatory Activities, system organ class and preferred terms (version 24.1).

#### 2.3.3 Pharmacokinetics and immunogenicity

Risankizumab serum concentrations, including antidrug antibodies (ADA) and neutralizing antibodies (NAb), were determined from blood samples collected through to week 68. The presence of ADA was assessed by a three‐tiered approach using an electrochemiluminescence assay; confirmed ADA‐positive samples were then evaluated for the presence of NAb. For the serum RZB pharmacokinetic assay, samples were collected at weeks 4, 16, 28, 52, 64, and 68; immunogenicity assay samples were collected at weeks 16, 28, 32, 52, and 68. The effects of immunogenicity on efficacy (change from baseline in PPPASI) at weeks 16 and 68 and safety (incidence of hypersensitivity and injection‐site reactions) were assessed.

### 2.4 Statistical analysis

A sample size of 116 patients was needed to provide greater than 95% power with a two‐sided significance level of 0.05 to detect a statistically significant difference in the primary end point between the RZB and placebo groups. Efficacy was analyzed in the intent‐to‐treat population, which included all randomized patients. For Period A, continuous end points (including physical activity analyses) were analyzed using a mixed‐effect model for repeated measures that included the fixed effects of treatment, visit, treatment‐by‐visit interaction, baseline smoking status, and baseline measurement as covariates. A mixed‐effect model for repeated measures was used to analyze longitudinal data and handle missing data for continuous data, with no special handling for data missing due to COVID‐19 infection or logistical restriction. Categorical secondary end points were analyzed using the Cochran–Mantel–Haenszel test stratified by baseline smoking status; missing data were imputed using non‐responder imputation incorporating multiple imputation to handle missing data due to COVID‐19 (i.e., multiple imputation was used to handle data missing due to COVID‐19 infection or logistical restriction; patients with missing data for any other reason were categorized as non‐responders using the traditional non‐responder imputation method). Efficacy in Period B was reported based on as‐observed data. Safety was analyzed in all randomized patients who received one or more doses of the study drug in each treatment period. The RZB data from all patients who received one or more doses of RZB were used to provide a comprehensive safety summary for RZB. Physical activity analyses were performed using the Python pymer4 to call the lme4 package in R.^23^, ^24^ All other analyses were performed using SAS statistical software version 9.4 (SAS Institute Inc.).

## 3 RESULTS

### 3.1 Patients

**FIGURE 1:**
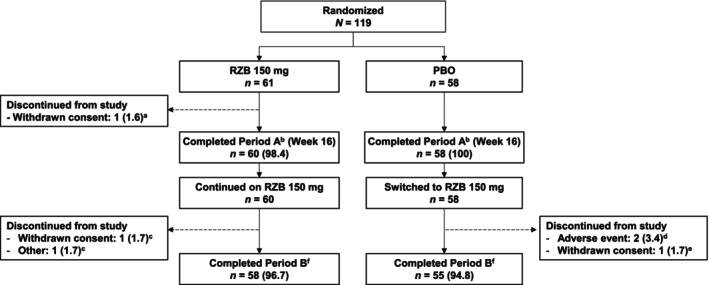
Patient disposition. Data are presented as *n* (%).^a^Discontinued due to patient‐reported physical and mental burden. ^b^Double‐blind period (weeks 0–16). ^c^Discontinued due to lack of efficacy. ^d^Discontinued due to alcoholic steatohepatitis (one patient) and hepatocellular carcinoma (one patient). ^e^Discontinued due to unrelated health concerns. ^f^Risankizumab (RZB)‐treatment period (weeks 16–68). PBO, placebo.

The JumPPP study was conducted from July 20, 2020, to November 21, 2022, at 39 study sites in Japan. Of 119 patients randomized to receive RZB (*n* = 61) or placebo (*n* = 58), 118 (99.2%) completed the double‐blind period; 113 (95.8%) of these completed the RZB‐treatment period (Figure 1). Baseline demographics and disease characteristics were generally similar across treatment groups (Table 1). Most patients were female (79.0%), and there were more female patients in the placebo group compared with the RZB group. Overall, 29.4% of patients with PPP had baseline PAO. Previous systemic therapy was reported in 55.5% of patients, including a history of previous biologic therapy in 1.7% of patients.

**TABLE 1:** Baseline demographics and characteristics.

Characteristic	RZB 150 mg *n* = 61	PBO *n* = 58	Total *N* = 119
Age, years, mean (SD)	54.4 (10.6)	56.4 (11.2)	55.4 (10.9)
Female, *n* (%)	44 (72.1)	50 (86.2)	94 (79.0)
Weight, kg, mean (SD)	64.9 (11.6)	63.1 (11.0)	64.0 (11.3)
BMI, kg/m^2^, mean (SD)	24.6 (3.5)	24.6 (4.0)	24.6 (3.7)
Disease duration, years, mean (SD)	7.8 (8.4)	6.8 (6.7)	7.3 (7.6)
Baseline smoking status, *n* (%)			
Yes	28 (45.9)	29 (50.0)	57 (47.9)
No	33 (54.1)	29 (50.0)	62 (52.1)
Prior systemic therapy, *n* (%)^a^	33 (54.1)	33 (56.9)	66 (55.5)
Biologic therapy, *n* (%)^b^	1 (1.6)	1 (1.7)	2 (1.7)
Corticosteroid use, *n* (%)	2 (3.3)	7 (12.1)	9 (7.6)
Immunosuppressant use, *n* (%)	15 (24.6)	8 (13.8)	23 (19.3)
Prior phototherapy, *n* (%)	23 (37.7)	26 (44.8)	49 (41.2)
PPPASI total score, mean (SD)	28.0 (10.2)	28.1 (10.6)	28.1 (10.4)
PAO, *n* (%)	19 (31.1)	16 (27.6)	35 (29.4)
mBASDAI^c^			
Mean (SD)	4.2 (2.6)	5.5 (2.5)	4.8 (2.6)
Median (range)	4.3 (0.8–9.5)	5.4 (0.7–9.6)	5.0 (0.7–9.6)
mBASDAI anterior chest pain subscore^c^			
Mean (SD)	3.9 (3.1)	4.4 (2.8)	4.1 (2.9)
Median (range)	2.5 (0–9.0)	4.5 (0–9.0)	3.5 (0–9.0)
WP‐NRS, mean (SD)	4.5 (2.9)	5.7 (2.9)	5.1 (2.9)
DLQI, mean (SD)	7.7 (5.2)	8.3 (5.6)	8.0 (5.4)

Abbreviations: BMI, body mass index; DLQI, Dermatology Life Quality Index; mBASDAI, modified Bath Ankylosing Spondylitis Disease Activity Index; PAO, pustulotic arthro‐osteitis; PBO, placebo; PPPASI, Palmoplantar Pustulosis Area and Severity Index; RZB, risankizumab; SD, standard deviation; WP‐NRS, worst pruritus numeric rating scale.

^a^ Including prior phototherapy.

^b^ All prior biologic therapy was guselkumab.

^c^ Among patients with PAO; RZB, *n* = 18; PBO, *n* = 16; total, *n* = 34.

### 3.2 Efficacy

#### 3.2.1 Change in PPPASI from baseline

**FIGURE 2:**
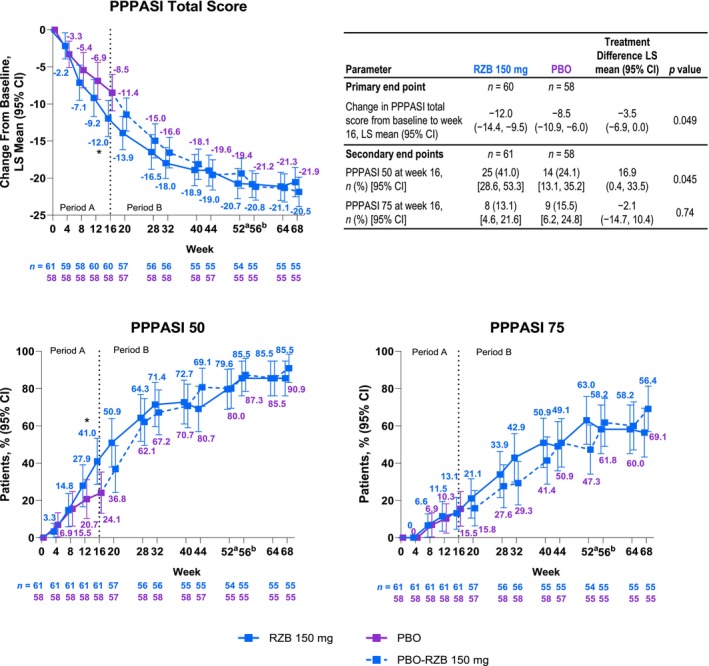
Palmoplantar Pustulosis Area and Severity Index (PPPASI) outcomes. For change from in PPPASI total score from baseline, a mixed‐effect model for repeated measures was used in the double‐blind period (Period A; weeks 0–16) and included the fixed effects of treatment, visit, treatment‐by‐visit interaction, baseline smoking status, and baseline measurement as covariates. For a ≥50% or ≥75% improvement in PPPASI (PPPASI 50/75), non‐responder imputation while incorporating multiple imputation to handle missing data due to COVID‐19 was used in the placebo‐controlled period (Period A; weeks 0–16); missing data due to COVID‐19 infection or logistical restriction were handled by multiple imputation. Observed‐cases analysis was used in the risankizumab (RZB)‐treatment period (Period B; weeks 16–68); observed‐cases analysis did not impute values for missing data, and patients missing data were excluded from the observed analysis for that visit. The dotted line represents the end of Period A. ^a^End of RZB treatment in RZB‐RZB arm. ^b^End of RZB treatment in the placebo (PBO)‐RZB arm. CI, confidence interval; LS, least squares. *p < 0.05 versus PBO; *p* values for PPPASI 50/75 are nominal.

At week 16, patients treated with RZB experienced greater improvement in signs and symptoms of PPP compared with placebo, as evidenced by achievement of the primary end point at week 16. RZB demonstrated significantly greater improvement over placebo in PPPASI total score change from baseline; the least squares (LS) mean (95% confidence interval [CI]) treatment difference was −3.48 (−6.94 to −0.02; *p* = 0.049) (Figure 2). After week 16, numerical improvements in the change in PPPASI total score from baseline were generally observed through to week 68 in the continuous RZB group. At week 68, the LS mean (95% CI) change from baseline in the continuous RZB group was −20.5 (−22.5 to −18.6). In the placebo‐RZB group, the trajectory change in PPPASI total score from baseline after week 16 was similar to that observed for patients randomized to RZB at the start of the study, with continued improvement over time to 68 weeks. The LS mean (95% CI) change from baseline in the placebo‐RZB group at week 68 was −21.9 (−23.9 to −19.9).

#### 3.2.2 PPPASI 50 and PPPASI 75

A numerically higher proportion of patients treated with RZB achieved PPPASI 50 versus placebo (41.0% vs 24.1%; nominal *p* = 0.045) at week 16; PPPASI 75 achievement at week 16 was similar between the RZB and placebo groups (13.1% vs 15.5%; nominal *p* = 0.74; Figure 2). Improvements in PPPASI 50 and PPPASI 75 responses were seen from weeks 16 to 68 in both the continuous RZB and placebo‐RZB groups.

#### 3.2.3 Patient‐reported outcomes

**FIGURE 3:**
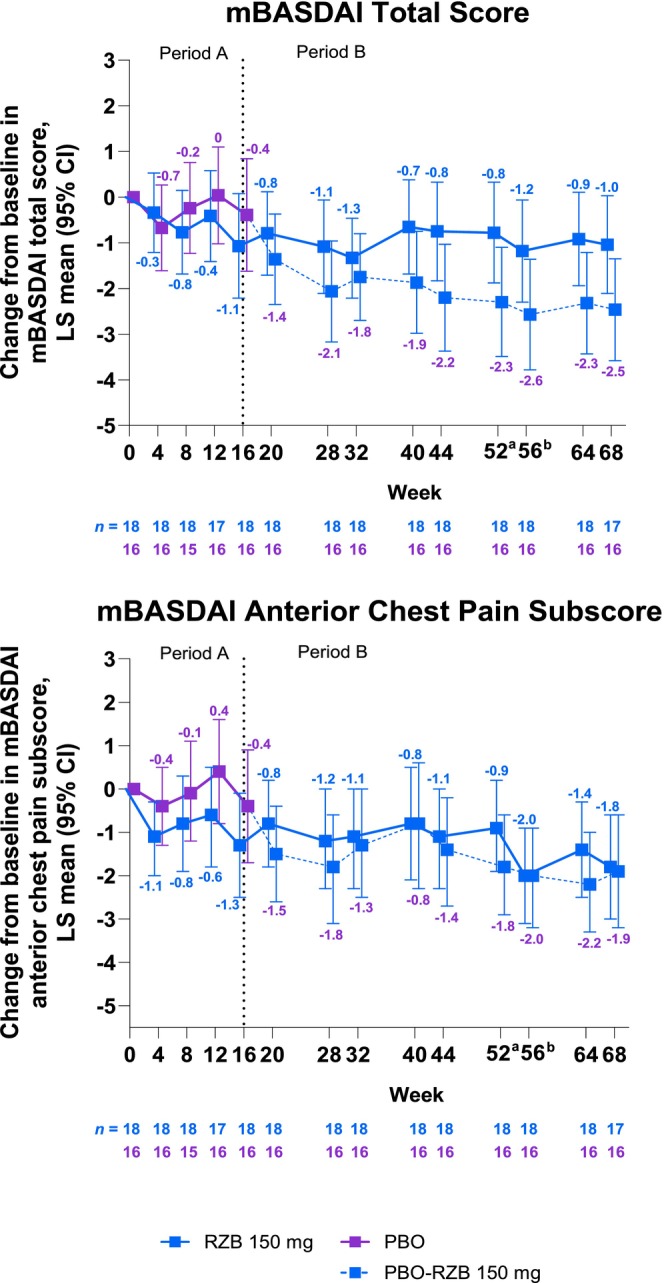
Change from baseline in modified Bath Ankylosing Spondylitis Disease Activity Index (mBASDAI) among patients with baseline pustulotic arthro‐osteitis (PAO). PAO diagnosis was confirmed by magnetic resonance imaging. A mixed‐effect model for repeated measures was used in the double‐blind period (Period A; weeks 0–16) and included the fixed effects of treatment, visit, treatment by visit interaction, baseline smoking status, and baseline measurement as covariates. Observed‐cases analysis was used in the risankizumab (RZB)‐treatment period (Period B; weeks 16–68); observed‐cases analysis did not impute values for missing data, and patients missing data were excluded from the observed analysis for that visit. The dotted line represents the end of Period A. ^a^End of RZB treatment in RZB‐RZB arm. ^b^End of RZB treatment in placebo (PBO)‐RZB arm. CI, confidence interval; LS, least squares.

Among patients with baseline PAO, numerically greater improvements in mBASDAI total score were seen in the RZB versus placebo group at week 16 (Figure 3); the LS mean (95% CI) treatment difference was −0.7 (−2.4 to 1.0). After week 16, improvements in mBASDAI total score were generally sustained to week 68 in the continuous RZB group. Up to week 68, patients in the placebo‐RZB group generally continued to improve in mBASDAI total score after the first RZB dose at week 16; numerically greater improvements in mBASDAI total score were observed in the placebo‐RZB versus the continued RZB group from weeks 20 to 68. For the mBASDAI anterior chest pain subscore, the RZB group also showed improvements from weeks 4 to 68 (Figure 3). Improvements in mBASDAI anterior chest pain subscore were observed in the placebo‐RZB group after switching to RZB at week 16 and were generally sustained to week 68.

**FIGURE 4:**
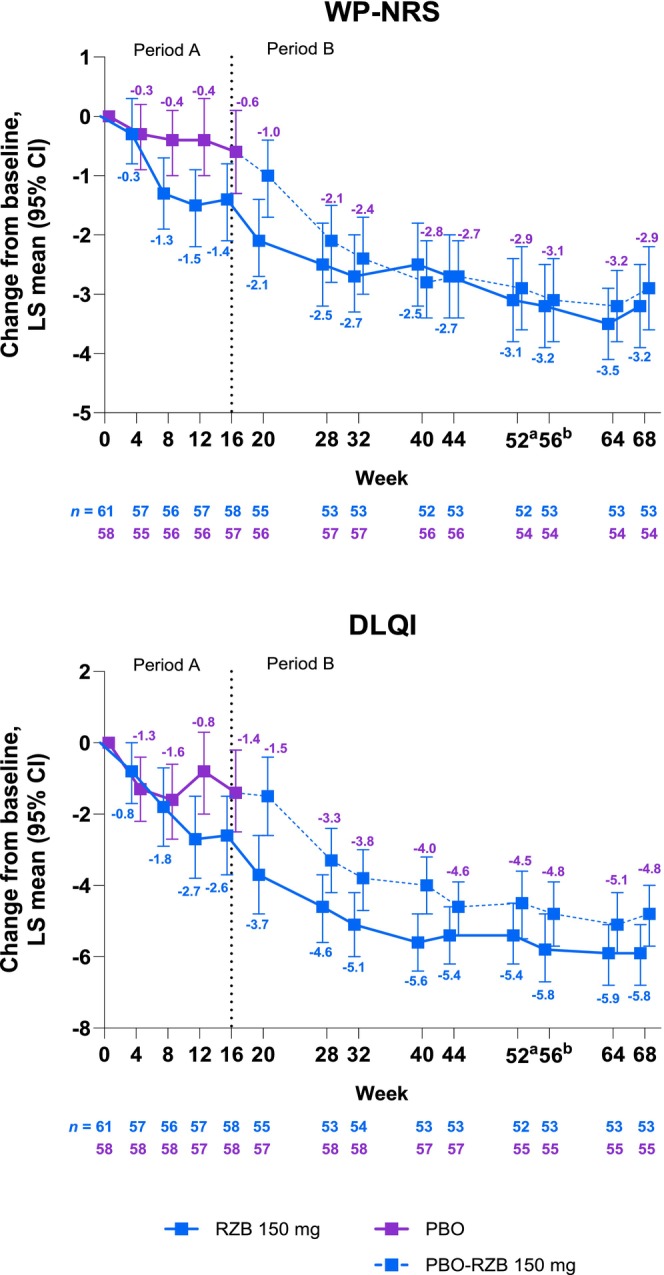
Change from baseline in worst pruritus numeric rating scale (WP‐NRS) and Dermatology Life Quality Index (DLQI). A mixed‐effect model for repeated measures was used in the double‐blind period (Period A; weeks 0–16) and included the fixed effect of treatment, visit, treatment by visit interaction, baseline smoking status, and baseline measurement as covariates. Observed‐cases analysis was used in the risankizumab (RZB)‐treatment period (Period B; weeks 16–68); observed‐cases analysis did not impute values for missing data, and patients missing data were excluded from the observed analysis for that visit. The dotted line represents the end of Period A. ^a^End of RZB treatment in RZB‐RZB arm. ^b^End of RZB treatment in placebo (PBO)‐RZB arm. CI, confidence interval; LS, least squares; PBO, placebo.

Greater improvements in itch reduction were observed in the RZB versus the placebo group, as shown by the numerically larger change from baseline in WP‐NRS scores (Figure 4). At week 16, the LS mean (95% CI) treatment difference for WP‐NRS was −0.8 (−1.8 to 0.1). The RZB group showed greater improvements at week 16 in health‐related quality of life (HRQL) outcomes versus the placebo group, as shown by the numerically larger change from baseline in DLQI scores (Figure 4). At week 16, the LS mean (95% CI) treatment difference for DLQI was −1.2 (−2.9 to 0.4). Numerical improvements in WP‐NRS and DLQI were generally observed in patient‐reported outcomes after week 16 (in patients who were initially randomized to receive RZB and in those initially randomized to receive placebo).

#### 3.2.4 Physical activity

Physical activity data were available from weeks 1 to 16 during Period A and from weeks 41 to 52 during Period B. Patients who were initially treated with RZB had greater improvements from baseline in physical activity compared with those who received placebo, as demonstrated by numerically greater percentage changes in daily step count and MVPA through to week 16 that were sustained to week 52 (Figure S1). Similar patterns in LS mean daily step count and MPVA were generally observed in exploratory analyses of subgroups of patients with baseline PAO (Figure S2) or without baseline PAO (Figure S3), and patients who were sedentary (i.e., taking ≤5000 daily steps) at baseline (overall, Figure S4; with PAO, Figure S5; and without PAO, Figure S6). Median physical activity and clinical outcome scores over time for the overall population, patients with PAO, and patients who were sedentary at baseline are shown in Tables S1–S3, respectively.

### 3.3 Safety

During the double‐blind period, the proportion of patients with TEAEs and exposure‐adjusted rates of TEAEs were generally similar between the RZB group (52.5%; 319.4 events [E]/100 patient‐years [PY]) and the placebo group (53.4%; 303.4 E/100 PY; Table 2). Rates of serious AEs, severe AEs, and AEs leading to study drug discontinuation were generally similar between both groups, with no meaningful differences between the RZB and placebo groups.

**TABLE 2:** Safety overview.

Parameter	Week 16				End of study	
	RZB 150 mg *n* = 61 PY = 19.1		PBO *n* = 58 PY = 17.8		All RZB *n* = 119 PY = 149.6	
	*n* (%)	E (E/100 PY)	*n* (%)	E (E/100 PY)	*n* (%)	E (E/100 PY)
Any AE	32 (52.5)	61 (319.4)	31 (53.4)	54 (303.4)	90 (75.6)	312 (208.6)
Serious AE	2 (3.3)	2 (10.5)	2 (3.4)	2 (11.2)	5 (4.2)^a^	6 (4.0)^a^
Severe AE	3 (4.9)	3 (15.7)	5 (8.6)	7 (39.3)	7 (5.9)^b^	7 (4.7)^b^
AE related to the study drug	4 (6.6)	5 (26.2)	5 (8.6)	6 (33.7)	14 (11.8)	15 (10.0)
AE leading to study drug discontinuation	2 (3.3)	3 (15.7)	0	0	5 (4.2)^c^	6 (4.0)^c^
Serious infection	1 (1.6)	1 (5.2)	1 (1.7)	1 (5.6)	1 (0.8)^d^	1 (0.7)^d^
MACE	0	0	0	0	0	0
Active tuberculosis	0	0	0	0	0	0
Malignancies	0	0	0	0	2 (1.7)	2 (1.3)
Serious hypersensitivity	0	0	0	0	1 (0.8)	1 (0.7)
Deaths	0	0	0	0	0	0

Abbreviations: AE, adverse event; E, event; MACE, major adverse cardiac event; PBO, placebo; PY, patient‐years; RZB, risankizumab.

^a^ Included cataracts, cholangitis, chronic eosinophilic rhinosinusitis, hepatocellular carcinoma, herpes zoster, and hypopharyngeal cancer.

^b^ Included cholangitis, chronic eosinophilic rhinosinusitis, hepatocellular carcinoma, herpes zoster, hypopharyngeal cancer, palmoplantar pustulosis, and periodontal disease.

^c^ Included hepatitis alcoholic, hepatocellular carcinoma, palmoplantar pustulosis, pustular psoriasis, rash papular, and wrist fracture.

^d^ Included herpes zoster.

Through to 76 weeks (end of study), patients in the all RZB‐treated population experienced 312 TEAEs (208.6 E/100 PY). The exposure‐adjusted rate for TEAEs leading to study drug discontinuation was low (4.0 E/100 PY). The most frequently reported TEAEs (occurring in ≥5% of patients) were pyrexia, nasopharyngitis, eczema, back pain, dermatitis contact, dental caries, and periodontitis (Table S4). Of the 26 patients who experienced pyrexia, 22 experienced events that were associated with the COVID‐19 vaccine. There were no cases of major adverse cardiovascular events, adjudicated anaphylactic reaction, active tuberculosis, opportunistic infections (excluding tuberculosis and herpes zoster), non‐melanoma skin cancer, or death. Two serious malignancy events (1.3 E/100 PY) occurred in the all RZB‐treated population, including one event of hepatocellular carcinoma (assessed as unrelated to the study drug by the investigator but which led to study drug discontinuation) and one event of hypopharyngeal cancer (assessed as related to the study drug by the investigator but did not lead to study drug discontinuation). The incidence of predefined hepatic events of safety interest was low (2.7 E/100PY); no events led to interruption or discontinuation of the study drug. Though data on concomitant conditions and incidence of focal infections were not formally collected, among the reported information, one patient with a known medical history of eosinophilic sinusitis experienced a serious AE of worsening eosinophilic sinusitis while taking blinded RZB; the event was treated and resolved 7 days later, was considered to be unrelated to RZB, and did not lead to study drug discontinuation.

### 3.4 Immunogenicity

Geometric mean RZB trough plasma concentrations were maintained at a similar level in each group from weeks 28 to 64 (continued RZB, range, 2.0–2.7 μg/mL; placebo‐RZB, range, 4.1–5.4 μg/mL). Approximately 11.7% (7/60) and 10.0% (6/60) of evaluable RZB‐treated patients developed treatment‐emergent ADA and NAb, respectively. The geometric mean serum RZB concentrations in ADA‐ or NAb‐positive patients were generally similar to or slightly lower than those for ADA‐ or NAb‐negative patients throughout the study. Similar mean changes from baseline in PPPASI total scores were observed for ADA/NAb‐positive and ADA/NAb‐negative patients at weeks 16 and 68 (Table S5). The incidence of hypersensitivity and injection site reactions to the end of the study were generally similar between ADA‐ or NAb‐positive patients when compared with ADA‐ or NAb‐negative patients (Table S6).

## 4 DISCUSSION

Results from the phase 3 JumPPP study demonstrated clinically meaningful efficacy with RZB across multiple disease domains. Treatment with RZB resulted in improved signs and symptoms of PPP. The primary end point of change in PPPASI total score from baseline at week 16 was achieved, as demonstrated by a statistically significant difference between the RZB and placebo groups. RZB treatment also resulted in greater response rates of PPPASI 50 (secondary end point) at week 16, while the PPPASI 75 response rate was similar between the RZB‐ and placebo‐treated groups. The disease characteristics of PPP often include repeated remission and relapse and a thickened horny layer of palmoplantar epidermis. Compared with erythema and vesicles, desquamation and scales (in which pustules/vesicles become crusted) take longer to resolve, and turnover of lesions takes time. As such, it may take a long time for a treatment to exert its therapeutic effect, and week 16 may have been too early to assess the PPPASI 75 response, which is closer to remission than a PPPASI 50 response. Variations due to the characteristics of PPP, including periods of improvement followed by exacerbations, may also partially explain why the PPPASI 75 response rate at week 16 did not tend to be higher in the RZB group than in the placebo group.

After week 16, efficacy for most outcomes was sustained or improved in patients who were initially randomized to RZB and in those initially randomized to receive placebo. The PPPASI 75 response rate generally continued to increase after week 16 in both groups, and, notably, the trajectory from week 16 to week 68 in the placebo‐to‐RZB group was similar to the first 52 weeks of treatment in the RZB group. These patterns show that patients may need to receive RZB for a longer period (i.e, more than 16 weeks) before achieving the more stringent, difficult‐to‐achieve PPPASI 75 end point. Overall, long‐term treatment with RZB demonstrated consistent efficacy through to 68 weeks in patients with PPP.

Patients with PPP experience reduced HRQL and significant daily functional physical impairment compared with patients with moderate‐to‐severe plaque psoriasis.^7^, ^8^ This is expected given the more extensive nature of the disease, in which patients with PPP experience greater restriction of the palms and soles that are crucial for activities of daily living. In this study, patients who received RZB reported improved HRQL at week 16, as indicated by DLQI improvements from baseline. Among patients with imaging‐confirmed baseline PAO, mBASDAI scores assessing joint symptoms (pain, swelling, and stiffness) in PPP generally improved from baseline to week 16 with RZB treatment; however, these results should be interpreted with caution given the small sample size of patients with a diagnosis of PAO at baseline. Additionally, most patients treated with RZB reported reduced itch symptoms, as assessed by a change from baseline in WP‐NRS. Patients also experienced increased physical activity at week 16 (as measured by daily step count and MVPA) with RZB treatment. Interestingly, patients who were sedentary at baseline and patients with baseline PAO showed numerically greater improvements in daily step count and MVPA compared with the overall population. This could be due to greater suppression of physical activity in patients with baseline PAO. However, among patients who were sedentary at baseline, greater improvements in physical activity with RZB over placebo were observed in the subgroup of patients without PAO at baseline. Though these data should be interpreted cautiously given the limited number of patients in each subgroup, this finding suggests skin improvements may have a greater impact on enhancing physical activity than joint symptoms. Patients in the placebo‐RZB group did not demonstrate improvements from baseline in daily step count and MVPA that were observed among patients initially randomized to RZB. This discrepancy could be explained by a few patients initially randomized to the RZB group who experienced considerable improvements in physical activity due to low baseline activity levels, which may have inflated the overall mean of the RZB group. Improvements in the efficacy variables for HRQL and physical function were generally observed through to week 68 in patients who received continued RZB and those who received placebo then switched to RZB at week 16.

A concern with biologic treatment is the development of ADA and NAb because the associated undesirable immune responses (e.g., loss of response and increased adverse effects) can ultimately alter the pharmacological and/or pharmacokinetic properties of the drug and impact overall drug efficacy and safety.^25^ The geometric mean serum RZB concentrations in ADA‐/NAb‐positive patients were similar to or slightly lower than those in ADA/NAb‐negative patients at most visits, indicating limited impact of immunogenicity on RZB exposure. Furthermore, similar efficacy (change in PPPASI total score from baseline) and safety (injection site reactions and hypersensitivity reactions) were observed to the end of the study, regardless of ADA/NAb status, indicating no impact of immunogenicity on the efficacy or safety of RZB.

Exacerbating factors, such as focal infection, may contribute to the worsening symptoms of PPP.^9^ Thus, the relationship between focal infection flare‐ups and PPP treatments is of special interest. Though the incidence of focal infection was not formally assessed, only one serious AE of focal infection was identified.

Risankizumab was generally well tolerated by patients with PPP, and no new safety signals were identified. The exposure‐adjusted rates of TEAEs at the end of the study, including those leading to discontinuation or safety interest, were low and consistent with rates observed in the double‐blind period. The safety profile of RZB observed through the RZB treatment period and follow‐up for 76 weeks was generally consistent with the safety profile of RZB observed to 304 weeks of treatment in patients with psoriasis.^26^ In addition, safety findings were generally consistent with long‐term treatment with other biologics studied in Japanese adults with PPP.^12^

A limitation of this study is the small sample size, which may overestimate the magnitude of efficacy and AEs. Also, the interpretation of efficacy and safety outcomes in Period B is limited by the lack of a placebo group. Lastly, this study was conducted during the COVID‐19 pandemic, and COVID‐19–related logistical restrictions resulted in missing data at one visit for five patients. Data missing due to COVID‐19 infection or related logistical restrictions were addressed using specific imputation methods implemented to handle COVID‐19‐related missing data. These methods are unlikely to have influenced the study's findings because the primary end point was analyzed using a mixed‐effect model repeated measurements method that does not differentiate missing data due to COVID‐19, and no PPPASI data were missing due to COVID‐19 at week 16 (consequently, the analysis was equivalent to the traditional non‐responder imputation analysis).

These comprehensive results from the JumPPP study showed clinically meaningful and durable efficacy of RZB over time in patients with PPP. Efficacy was achieved at week 16 and generally continued to improve or be maintained throughout treatment and up to the last follow‐up visit. The safety profile was consistent with the safety profiles reported in other RZB studies in psoriasis. Overall, these results suggest that RZB may be an effective long‐term treatment option for the effective management of PPP.

## CONFLICT OF INTEREST STATEMENT

Yukari Okubo has received research funds from AbbVie, Eisai, Maruho, Shiseido, Sun Pharma, and Torii. She has received honoraria for speaking, consultancy, and as an advisory board member from AbbVie, Amgen, Boehringer Ingelheim, Bristol Myers Squibb, Eli Lilly, Janssen, Kyowa Kirin, LEO Pharma, Maruho, Novartis, Pfizer, Sanofi, Sun Pharma, Taiho, and UCB Japan. She has received honoraria for speaking from Eisai, Jimro, Mitsubishi Tanabe, and Torii. Masamoto Murakami has received research support and/or served as a consultant for AbbVie, Amgen, Aristea Therapeutics, Boehringer Ingelheim, Bristol Myers Squibb, Eisai, Eli Lilly, Janssen, Kyowa Kirin, and Novartis. His current affiliations are with Atsuta Skin Clinic, Nagoya, Japan, and the Department of Anatomy, Histochemistry, and Cell Biology, Faculty of Medicine, University of Miyazaki, Miyazaki, Japan. Satomi Kobayashi has received research grants from Kyowa Kirin. She has received honoraria from AbbVie, Amgen, Eli Lilly, Janssen, Maruho, Novartis, and Taiho. Shigeyoshi Tsuji has received consulting fees and/or honoraria from AbbVie, Asahi Kasei Pharma, Daiichi Sankyo, Eisai, Eli Lilly, Janssen, Kyowa Kirin, Taiho, and UCB. Mitsumasa Kishimoto has received consulting fees and/or honoraria from AbbVie, Amgen, Asahi Kasei Pharma, Astellas, Ayumi Pharma, Bristol Myers Squibb, Chugai, Daiichi‐Sankyo, Eisai, Eli Lilly, Gilead, Janssen, Mitsubishi Tanabe, Novartis, Ono Pharma, Pfizer, and UCB. Kimitoshi Ikeda, Maiko Jibiki Ezequiel Neimark, Byron Padilla, and Jie Shen are full‐time employees of AbbVie Inc. or AbbVie GK, and may hold AbbVie stock or stock options. Sydney Peters is a former employee of AbbVie and may hold AbbVie stock. Tadashi Terui reports grants and/or personal fees from AbbVie, Boehringer Ingelheim, Eisai, Eli Lilly, Janssen, Kaken Pharmaceutical, Kyorin, Kyowa Kirin, Maruho, Mitsubishi Tanabe, Nihon Pharmaceutical, Novartis, Sanofi, Sun Pharma, and Taiho.

## ETHICS STATEMENT

This clinical study was conducted in accordance with the protocol of the International Council for Harmonization of Technical Requirements for Pharmaceuticals for Human Use guidelines and applicable guidelines and regulations governing ethical principles originating in the Declaration of Helsinki. An independent ethics committee/institutional review board ensured the ethical, scientific, and medical appropriateness of the study before it was conducted and approved all relevant documentation. Written informed consent was obtained from all patients before enrollment. This clinical study was prospectively registered at ClinicalTrials.gov (NCT04451720).

## Supporting information


Data S1.

## Data Availability

AbbVie is committed to responsible data sharing regarding the clinical trials we sponsor. This includes access to anonymized individual and trial‐level data (analysis data sets), as well as other information (e.g., protocols, clinical study reports, or analysis plans), as long as the trials are not part of an ongoing or planned regulatory submission. This includes requests for clinical trial data for unlicensed products and indications. These clinical trial data can be requested by any qualified researchers who engage in rigorous, independent scientific research, and will be provided following review and approval of a research proposal and statistical analysis plan and execution of a data sharing agreement. Data requests can be submitted at any time after approval in the United States and Europe and after acceptance of this manuscript for publication. These data will be accessible for 12 months, with possible extensions considered. For more information on the process or to submit a request, visit the following link: https://vivli.org/ourmember/abbvie/ then select “Home.”
