# Supplementary material for: Risankizumab in Japanese patients with moderate‐to‐severe palmoplantar pustulosis: Results from the randomized, phase 3 JumPPP study
Source: J Dermatol. 2025 Feb 25;52(4):593–602. doi: 10.1111/1346-8138.17659 (PMC11975171; doi:10.1111/1346-8138.17659)

Risankizumab in Japanese patients with moderate-to-severe palmoplantar pustulosis: Results from the randomized, phase 3 JumPPP study

SUPPORTING INFORMATION

Yukari Okubo[1] | Masamoto Murakami[2] | Satomi Kobayashi[3] | Shigeyoshi Tsuji[4] | Mitsumasa Kishimoto[5] | Kimitoshi Ikeda[6] | Maiko Jibiki[6] | Ezequiel Neimark[7] | Byron Padilla[7] | Jie Shen[7] | Sydney Peters[7] | Tadashi Terui[8]

[1]Department of Dermatology, Tokyo Medical University Hospital, Tokyo, Japan
[2]Department of Dermatology, Ehime University School of Medicine, Ehime, Japan
[3]Department of Dermatology, Seibo International Catholic Hospital, Tokyo, Japan
[4]Department of Rehabilitation, Orthopedics and Psoriasis Center, Nippon Life Hospital, Osaka, Japan
[5]Department of Nephrology and Rheumatology, Kyorin University School of Medicine, Tokyo, Japan
[6]AbbVie GK, Tokyo, Japan
[7]AbbVie Inc., North Chicago, Illinois, USA
[8]Division of Dermatological Science, Department of Dermatology, Nihon University School of Medicine, Tokyo, Japan

Correspondence
Yukari Okubo
Department of Dermatology
Tokyo Medical University Hospital
6-7-1 Nishishinjuku, Shinjuku-ku
Tokyo 160-0023, Japan
Email: yukari-o@tokyo-med.ac.jp
Phone: 03-5339-3136
Fax: 03-3342-3535
ORCID: 0000-0002-9526-1259

TABLE S1 Median physical activity and clinical outcome scores (overall population).

Timepoint Group	Daily step count	MVPA, min/d	PPPASI total score	mBASDAI total score[a]	mBASDAI anterior chest pain subscore[a]
Baseline					
RZB	*n* = 59<br>5043	*n* = 59<br>94.3	*n* = 61<br>26.7	*n* = 18<br>4.3	*n* = 18<br>2.5
PBO	*n* = 56<br>4942	*n* = 56<br>103.3	*n* = 58<br>28.0	*n* = 16<br>5.4	*n* = 16<br>4.5
Week 16					
RZB	*n* = 58<br>4214	*n* = 58<br>92.4	*n* = 58<br>14.7	*n* = 18<br>2.6	*n* = 18<br>2.0
PBO	*n* = 55<br>4988	*n* = 55<br>105.8	*n* = 58<br>18.1	*n* = 16<br>4.2	*n* = 16<br>3.5
Week 40/41					
RZB	*n* = 50<br>4478	*n* = 50<br>92.9	*n* = 55<br>6.8	*n* = 18<br>3.2	*n* = 18<br>3.0
PBO/RZB	*n* = 50<br>4771	*n* = 50<br>104.6	*n* = 58<br>9.3	*n* = 16<br>2.6	*n* = 16<br>2.0
Week 52					
RZB	*n* = 50<br>4388	*n* = 50<br>100.1	*n* = 54<br>3.8	*n* = 18<br>2.1	*n* = 18<br>2.0
PBO/RZB	*n* = 46<br>5028	*n* = 46<br>103.9	*n* = 55<br>6.6	*n* = 16<br>2.3	*n* = 16<br>1.5

Abbreviations: mBASDAI, modified Bath Ankylosing Spondylitis Disease Activity Index; MVPA, moderate-to-vigorous physical activity; PBO, placebo; PPPASI, Palmoplantar Pustulosis Area and Severity Index; RZB, risankizumab.
All data are median values for observed cases.
Week 40 data presented for PPASI total score, mBASDAI total score, and mBASDAI anterior chest pain subscore; week 41 data presented for daily step count and MVPA.
[a]Evaluated in patients with baseline pustulotic arthro-osteitis.

TABLE S2 Median physical activity and clinical outcome scores among patients with PAO.

Timepoint Group	Daily step count	MVPA, min/d	PPPASI total score	mBASDAI total score	mBASDAI anterior chest pain subscore
Baseline					
RZB	*n* = 18<br>2946	*n* = 18<br>69.1	*n* = 19<br>24.6	*n* = 18<br>4.3	*n* = 18<br>2.5
PBO	*n* = 16<br>5038	*n* = 16<br>87.6	*n* = 16<br>28.0	*n* = 16<br>5.4	*n* = 16<br>4.5
Week 16					
RZB	*n* = 18<br>3674	*n* = 18<br>90.1	*n* = 19<br>13.2	*n* = 18<br>2.6	*n* = 18<br>2.0
PBO	*n* = 16<br>4387	*n* = 16<br>100.5	*n* = 16<br>20.7	*n* = 16<br>4.2	*n* = 16<br>3.5
Week 40/41					
RZB	*n* = 17<br>3600	*n* = 17<br>83.0	*n* = 19<br>5.6	*n* = 18<br>3.2	*n* = 18<br>3.0
PBO/RZB	*n* = 13<br>3084	*n* = 13<br>83.6	*n* = 16<br>6.8	*n* = 16<br>2.6	*n* = 16<br>2.0
Week 52					
RZB	*n* = 17<br>4108	*n* = 17<br>88.7	*n* = 19<br>3.6	*n* = 18<br>2.1	*n* = 18<br>2.0
PBO/RZB	*n* = 14<br>3257	*n* = 14<br>82.3	*n* = 16<br>6.8	*n* = 16<br>2.3	*n* = 16<br>1.5

Abbreviations: mBASDAI, modified Bath Ankylosing Spondylitis Disease Activity Index; MVPA, moderate-to-vigorous physical activity; PAO, pustulotic arthro-osteitis; PBO, placebo; PPPASI, Palmoplantar Pustulosis Area and Severity Index; RZB, risankizumab.
All data are median values.
Week 40 data presented for PPASI total score, mBASDAI total score, and mBASDAI anterior chest pain subscore; week 41 data presented for daily step count and MVPA.

TABLE S3 Median physical activity and clinical outcome scores among patients taking ≤5000 daily steps at baseline.

Timepoint Group	Daily step count	MVPA, min/d	PPPASI total score	mBASDAI total score[a]	mBASDAI anterior chest pain subscore[a]
Baseline					
RZB	*n* = 29<br>2766	*n* = 29<br>67.2	*n* = 29<br>31.2	*n* = 13<br>4.6	*n* = 13<br>7.0
PBO	*n* = 31<br>3092	*n* = 31<br>79.0	*n* = 32<br>27.7	*n* = 8<br>5.9	*n* = 8<br>5.5
Week 16					
RZB	*n* = 31<br>3118	*n* = 31<br>80.6	*n* = 28<br>13.8	*n* = 13<br>2.9	*n* = 13<br>2.0
PBO	*n* = 29<br>3194	*n* = 29<br>97.7	*n* = 32<br>17.8	*n* = 8<br>6.3	*n* = 8<br>6.0
Week 40/41					
RZB	*n* = 25<br>3422	*n* = 25<br>89.0	*n* = 26<br>6.1	*n* = 13<br>2.3	*n* = 13<br>3.0
PBO/RZB	*n* = 29<br>3164	*n* = 29<br>83.0	*n* = 32<br>9.3	*n* = 8<br>4.1	*n* = 8<br>3.0
Week 52					
RZB	*n* = 24<br>3167	*n* = 24<br>90.6	*n* = 25<br>3.4	*n* = 13<br>1.9	*n* = 13<br>2.0
PBO/RZB	*n* = 24<br>2730	*n* = 24<br>84.0	*n* = 30<br>6.9	*n* = 8<br>4.7	*n* = 8<br>2.5

Abbreviations: mBASDAI, modified Bath Ankylosing Spondylitis Disease Activity Index; MVPA, moderate-to-vigorous physical activity; PBO, placebo; PPPASI, Palmoplantar Pustulosis Area and Severity Index; RZB, risankizumab.
All data are median values.
Week 40 data presented for PPASI total score, mBASDAI total score, and mBASDAI anterior chest pain subscore; week 41 data presented for daily step count and MVPA.
[a]Evaluated in patients with baseline pustulotic arthro-osteitis.

TABLE S4 Most frequently reported TEAEs.

**TEAE, *n* (%)**	**All RZB *N* = 119**
Pyrexia[a]	26 (21.8)
Nasopharyngitis	11 (9.2)
Eczema	9 (7.6)
Back pain	7 (5.9)
Dermatitis contact	7 (5.9)
Dental caries	6 (5.0)
Periodontitis	6 (5.0)

Abbreviations: RZB, risankizumab; TEAE, treatment-emergent adverse event.
[a]Most patients (*n* = 22) had events associated with COVID-19 vaccination.

TABLE S5 Change in PPPASI total score from baseline by ADA status.

PPPASI total score, LS mean (95% CI)	Week 16		Week 52		Week 64		Week 68	
	**RZB**	**PBO**	**RZB/RZB**	**PBO/RZB**	**RZB/RZB**	**PBO/RZB**	**RZB/RZB**	**PBO/RZB**
ADA-positive	*n* = 7<br>−11.0<br>(−16.5, −5.5)	*n* = 5<br>−6.2<br>(−13.2, 0.8)	*n* = 7<br>−22.3<br>(−26.7, −17.8)	*n* = 4<br>−22.4<br>(−28.7, −16.2)	*n* = 7<br>−22.3<br>(−29.5, −15.1)	*n* = 4<br>−18.3<br>(−28.3, −8.3)	*n* = 7<br>−22.3<br>(−26.9, −17.7)	*n* = 4<br>−22.0<br>(−26.4, −13.7)
ADA-negative	*n* = 51<br>−12.1<br>(−14.7, −9.5)	*n* = 53<br>−8.6<br>(−11.2, −6.1)	*n* = 47<br>−20.4<br>(−22.7, −18.2)	*n* = 51<br>−19.2<br>(−21.4, −17.1)	*n* = 48<br>−21.0<br>(−23.1, −19.0)	*n* = 51<br>−21.5<br>(−23.5, −19.5)	*n* = 48<br>−20.3<br>(−22.5, −18.2)	*n* = 51<br>−22.1<br>(−24.2, −20.0)
NAb-positive	*n* = 6<br>−10.4<br>(−19.7, −1.0)	*n* = 2<br>−4.8<br>(−22.3, 12.7)	*n* = 6<br>−20.0<br>(−25.9, −14.2)	*n* = 2<br>−21.0<br>(−32.0, −10.0)	*n* = 6<br>−20.3<br>(−27.1, −13.6)	*n* = 2<br>−16.3<br>(−28.9, −3.7)	*n* = 6<br>−20.4<br>(−24.6, −16.3)	*n* = 2<br>−21.6<br>(−29.4, 13.8)
NAb-negative	*n* = 52<br>−12.1<br>(−14.7, −9.6)	*n* = 56<br>−8.6<br>(−11.1, −6.1)	*n* = 48<br>−20.8<br>(−23.0, −18.6)	*n* = 53<br>−19.4<br>(−21.5, −17.2)	*n* = 49<br>−21.4<br>(−23.4, −19.3)	*n* = 53<br>−21.4<br>(−23.3, −19.4)	*n* = 49<br>−20.6<br>(−22.8, −18.4)	*n* = 53<br>−21.9<br>(−23.9, −19.8)

Abbreviations: ADA, anti-drug antibody; CI, confidence interval; LS, least squares; NAb, neutralizing antibody; PBO, placebo; PPPASI, Palmoplantar Pustulosis Area and Severity Index; RZB, risankizumab.

TABLE S6 Hypersensitivity reaction and injection-site reaction by ADA status.

	Week 16 *n* (%)				End of Study *n* (%)	
	Hypersensitivity reaction[a]		Injection site reaction		Hypersensitivity reaction[a]	Injection site reaction
**TEAE, *n*/*n* (%)**	**RZB**	**PBO**	**RZB**	**PBO**	**All RZB**	
ADA-positive	0/3	0/0	0/3	0/0	3/12 (25.0)	1/12 (8.3)
ADA-negative	6/58 (10.3)	7/58 (12.1)	0/58	0/58	23/107 (21.5)	5/107 (4.7)
NAb-positive	0/1	0/0	0/1	0/0	1/8 (12.5)	1/8 (12.5)
NAb-negative	6/60 (10.0)	7/58 (12.1)	0/60	0/58	25/111 (22.5)	5/111 (4.5)

Abbreviations: ADA, anti-drug antibody; LS, least squares; NAb, neutralizing antibody; PBO, placebo; RZB, risankizumab; TEAE, treatment-emergent adverse event.

[a]Reported treatment-emergent hypersensitivity reactions included allergic conjunctivitis, allergic rhinitis, chronic eosinophilic rhinosinusitis, contact dermatitis, dermatitis, drug eruption, eczema, hand dermatitis, idiopathic urticaria, nummular eczema, scrotal dermatitis, and urticaria.

FIGURE S1 Overall percent change from baseline in physical activity. A mixed-effect model for repeated measures model was used and included the fixed effects of treatment, visit, treatment-by-visit interaction, baseline smoking status, and baseline measurement as covariates. The dotted line represents the end of Period A. CI, confidence interval; LS, least squares; MVPA, moderate-to-vigorous physical activity; PBO, placebo; RZB, risankizumab. *$p \le 0.05$; **$p \le 0.01$; ***$p \le 0.001$ vs PBO (weeks 1–16) or PBO-RZB (weeks 41–52).

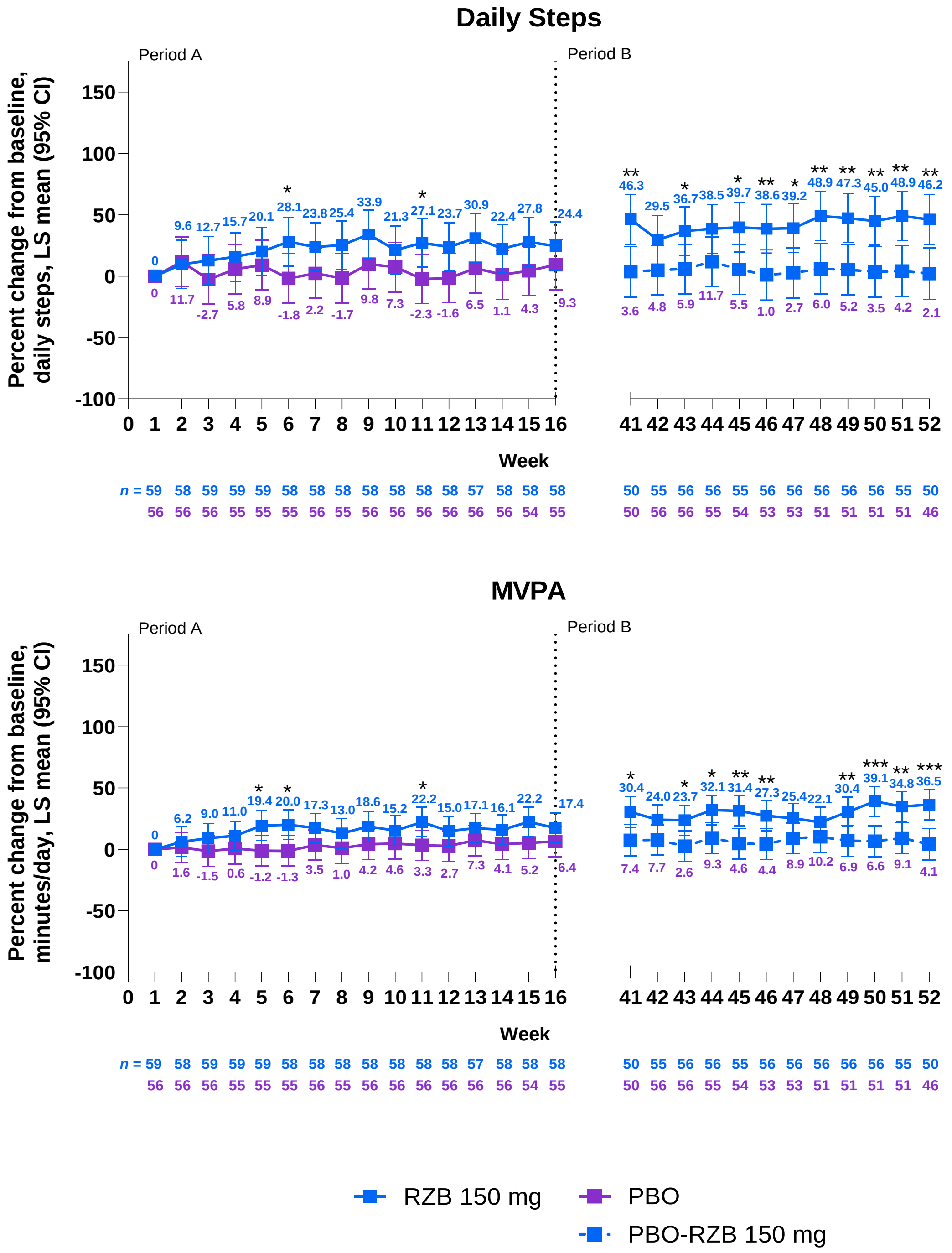

FIGURE S2 Percent change from baseline in physical activity among patients with PAO. A mixed-effect model for repeated measures was used and included the fixed effects of treatment, visit, treatment-by-visit interaction, baseline smoking status, and baseline measurement as covariates. The dotted line represents the end of Period A. CI, confidence interval; LS, least squares; MVPA, moderate-to-vigorous physical activity; PAO, pustulotic arthro-osteitis; PBO, placebo; RZB, risankizumab. *$p \le 0.05$ vs PBO (weeks 1–16) or PBO-RZB (weeks 41–52).

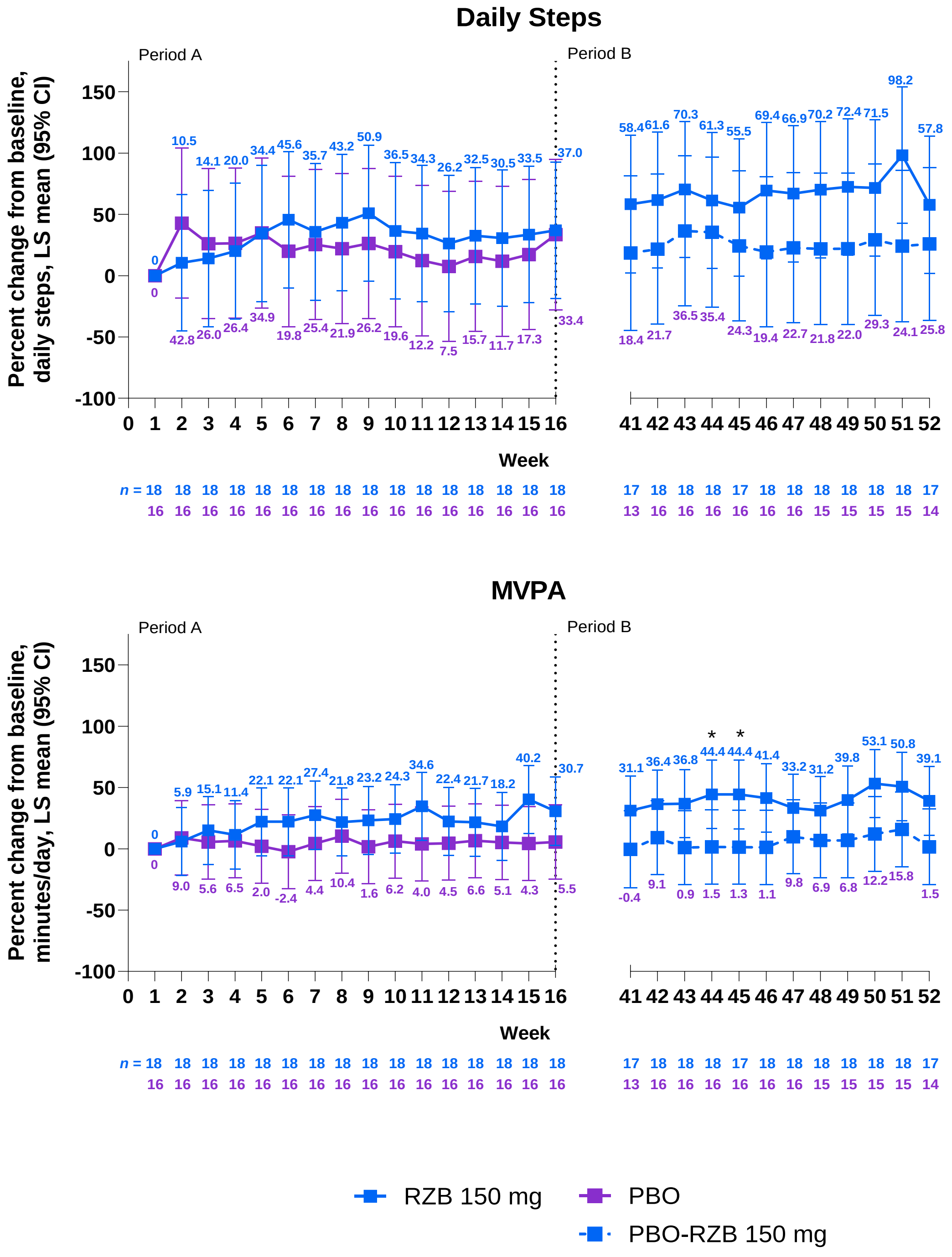

FIGURE S3 Percent change from baseline in physical activity among patients without PAO. A mixed-effect model for repeated measures was used and included the fixed effects of treatment, visit, treatment-by-visit interaction, baseline smoking status, and baseline measurement as covariates. The dotted line represents the end of Period A. CI, confidence interval; LS, least squares; MVPA, moderate-to-vigorous physical activity; PAO, pustulotic arthro-osteitis; PBO, placebo; RZB, risankizumab. **p* ≤ 0.05; ***p* ≤ 0.01; ****p* ≤ 0.001 vs PBO (weeks 1–16) or PBO-RZB (weeks 41–52).

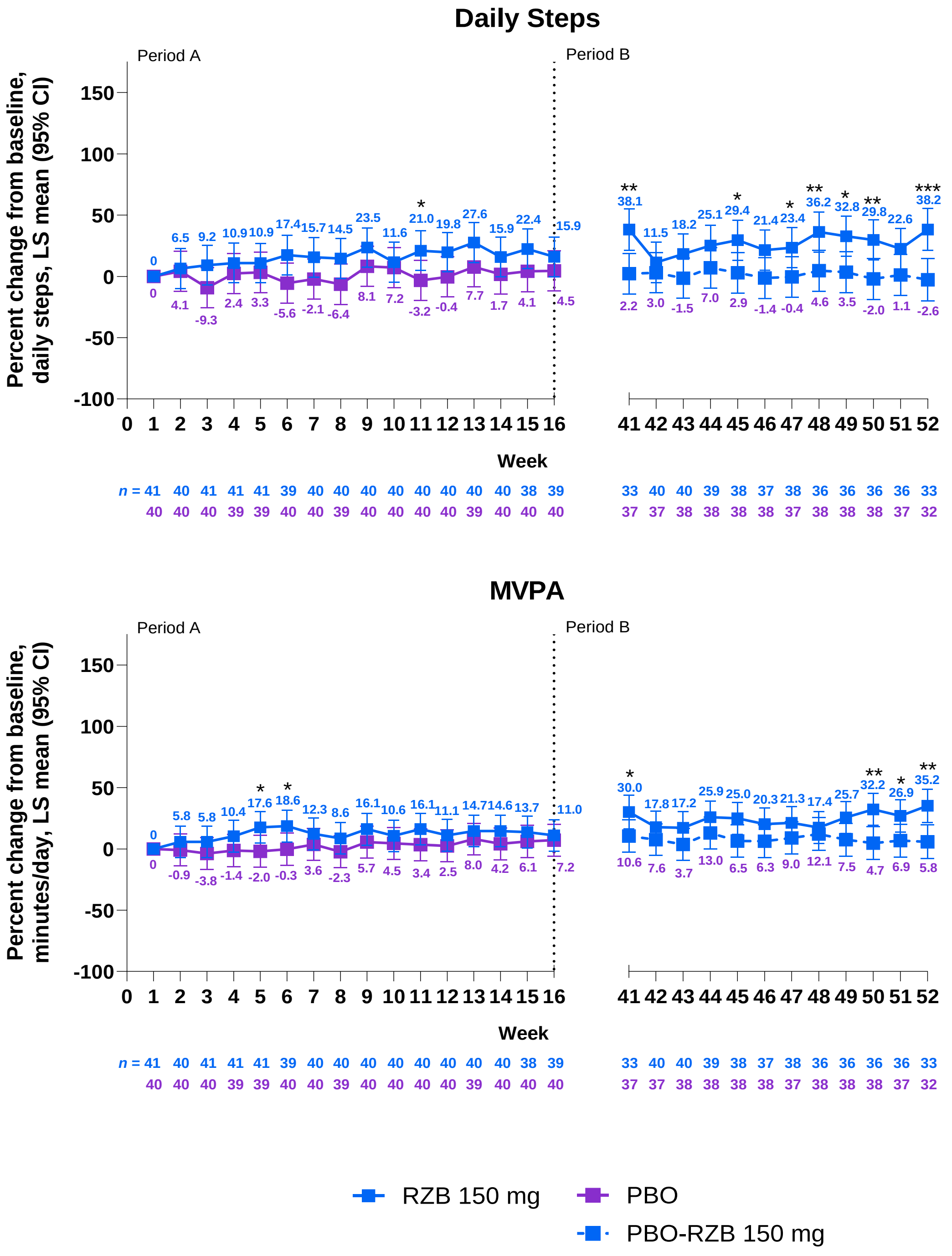

FIGURE S4 Percent change from baseline in physical activity among patients taking ≤5000 daily steps at baseline. A mixed-effect model for repeated measures was used and included the fixed effects of treatment, visit, treatment-by-visit interaction, baseline smoking status, and baseline measurement as covariates. The dotted line represents the end of Period A. CI, confidence interval; LS, least squares; MVPA, moderate-to-vigorous physical activity; PBO, placebo; RZB, risankizumab. *$p \leq 0.05$; **$p \leq 0.01$; ***$p \leq 0.001$ vs PBO (weeks 1–16) or PBO-RZB (weeks 41–52).

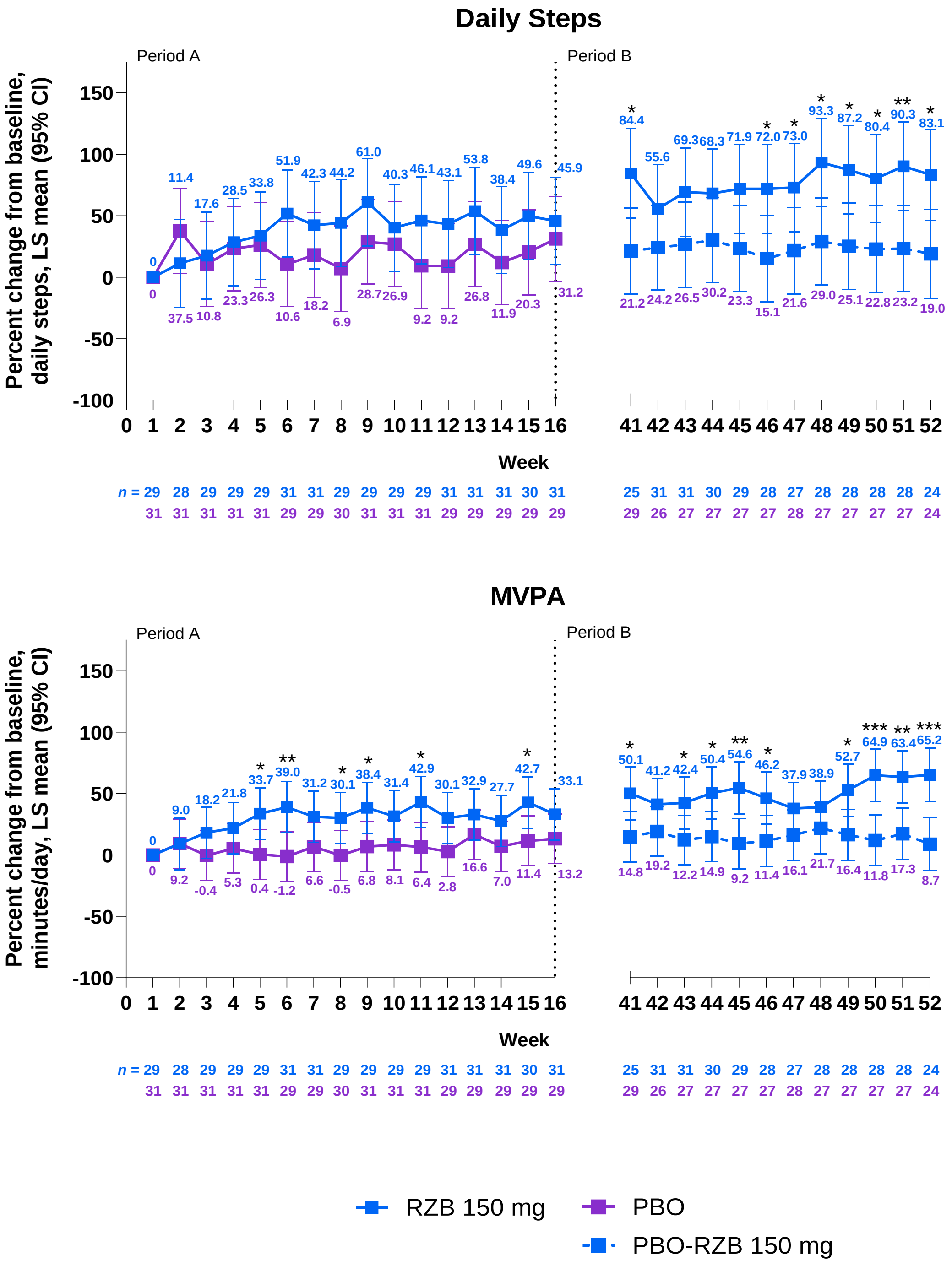

FIGURE S5. Percent change from baseline in physical activity among patients with PAO taking ≤5000 daily steps at baseline. A mixed-effect model for repeated measures was used and included the fixed effects of treatment, visit, treatment-by-visit interaction, baseline smoking status, and baseline measurement as covariates. The dotted line represents the end of Period A. CI, confidence interval; LS, least squares; MVPA, moderate-to-vigorous physical activity; PAO, pustulotic arthro-osteitis; PBO, placebo; RZB, risankizumab.

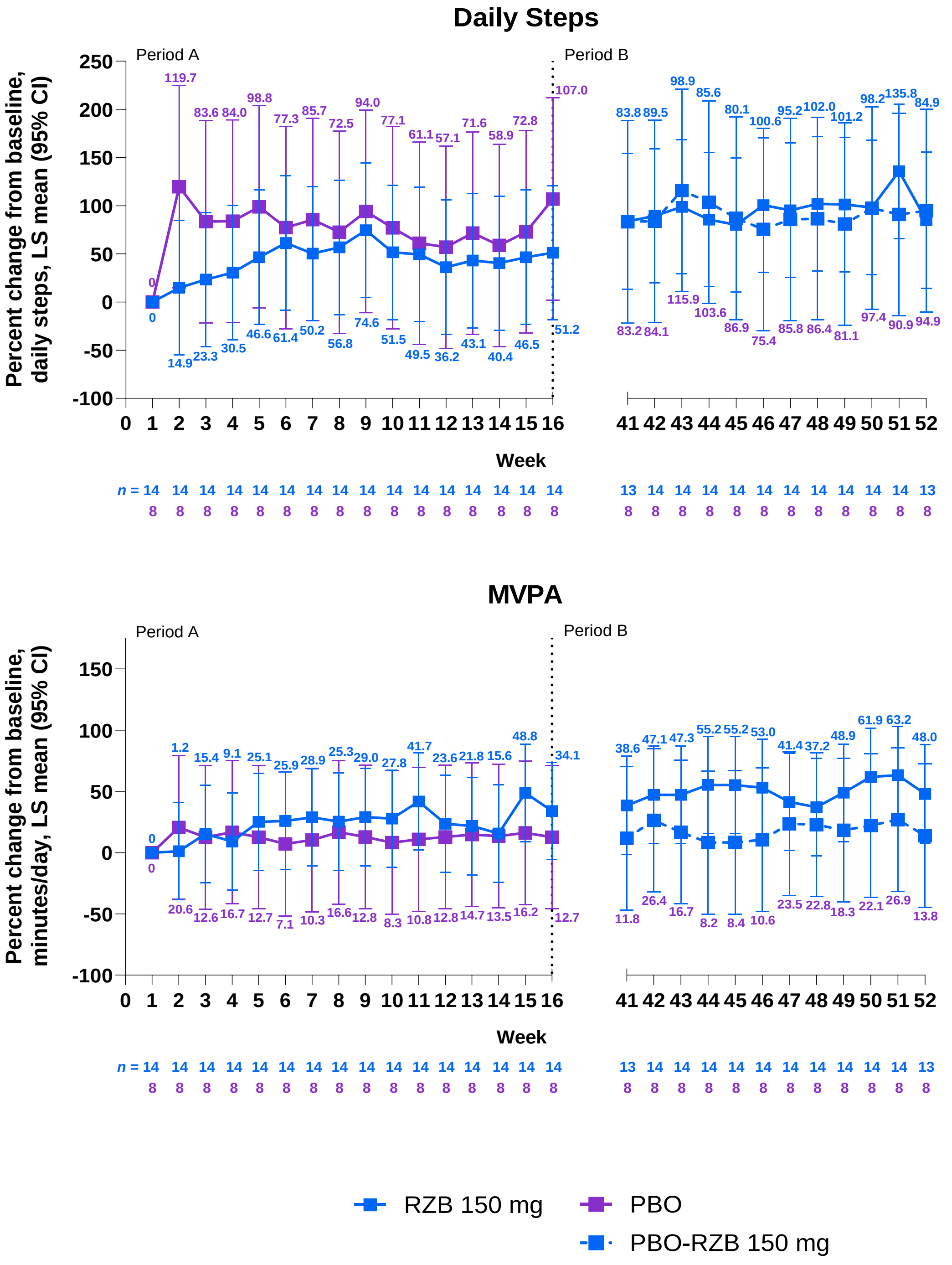

FIGURE S6. Percent change from baseline in physical activity among patients without PAO taking ≤5000 daily steps at baseline. A mixed-effect model for repeated measures was used and included the fixed effects of treatment, visit, treatment-by-visit interaction, baseline smoking status, and baseline measurement as covariates. The dotted line represents the end of Period A. CI, confidence interval; LS, least squares; MVPA, moderate-to-vigorous physical activity; PAO, pustulotic arthro-osteitis; PBO, placebo; RZB, risankizumab. *$p \leq 0.05$; **$p \leq 0.01$; ***$p \leq 0.001$ vs PBO (weeks 1–16) or PBO-RZB (weeks 41–52).

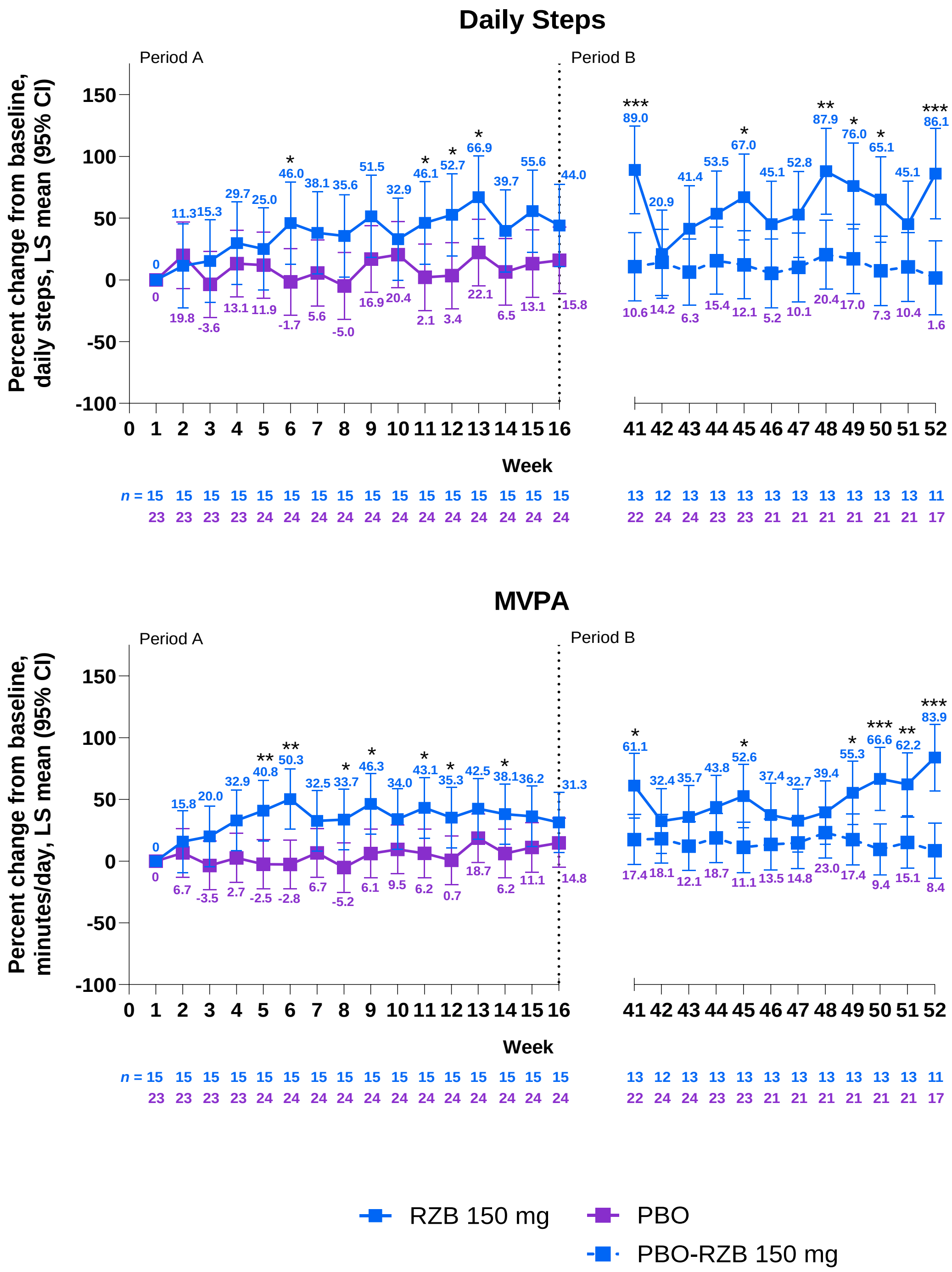

Supplement: Supplementary file 1 — Data S1. [file JDE-52-593-s001.pdf]
